# Estimating COVID-19 vaccine acceptance in pregnant and lactating women: a cross-sectional study in Lebanon

**DOI:** 10.1186/s13690-024-01267-8

**Published:** 2024-03-18

**Authors:** Dana Zayoud, Chadia Haddad, Dalia Khachman, Roula Ajrouche, Nathalie Lahoud

**Affiliations:** 1 Clinical and Epidemiological Research Laboratory, Faculty of Pharmacy, Lebanese University, Beirut, Lebanon; 2 Institut National de Santé Publique, d’Épidémiologie Clinique et de Toxicologie-Liban (INSPECT-LB), Beirut, Lebanon; 3 Faculty of Public Health, Lebanese University, Fanar, Lebanon; 4 School of Medicine, Lebanese American University, Byblos, Lebanon; 5 Research Department, Psychiatric Hospital of the Cross, P.O. Box 60096, Jal Eddib, Lebanon; 6 Department of Health Sciences, University of Sciences and Arts in Lebanon, Beirut, Lebanon

**Keywords:** Pregnant and breastfeeding women, Attitude, Vaccination, COVID-19, Risk, Acceptance, Reluctance

## Abstract

**Background:**

The COVID-19 pandemic highlights vaccination’s critical role in reducing morbidity and mortality, depending on public attitude. This study aims to identify the estimates of COVID-19 vaccine acceptance in pregnant and lactating women, as well as associated potential factors.

**Methods:**

A cross-sectional study was conducted between August and September 2021, through an online survey and with a paper survey distributed in gynecology and pediatric clinics. Pregnant and breastfeeding women aged 18 years and above were recruited. The attitude scale was created specifically for evaluating attitudes towards the COVID-19 vaccine.

**Results:**

In total, 207 women participated, with 132 breastfeeding, 74 pregnant and 1 experiencing both conditions. Of these, one hundred and twenty women (58%) considered themselves at risk for COVID-19 infection. In addition, 51.7% (*n* = 107) of women expressed the intent to receive the vaccine once available. A multivariable linear regression was conducted taking the COVID-19 vaccination attitude scale as a dependent variable. The results revealed an R-squared value of 0.558, indicating that approximately 55.8% of the variance in the attitude scale was accounted for by the included predictors. The results showed that preventive measures (ß=2.25, 95% Confidence Interval (CI) [1.02; 3.48], *p* < 0.001), preference for vaccines made in Europe and America (ß=1.23; 95% CI [0.69–1.77], *p* < 0.001), protect yourself for getting sick (ß=4.22, 95% Confidence Interval (CI) [2.83; 5.61], *p* < 0.001) and belief in the importance of vaccination for themselves and their baby (ß=3.49; 95% CI [2.01; 4.98], *p* < 0.001) were associated with a positive attitude towards vaccination. Conversely, experiencing a previous bad reaction to a vaccine (ß= -1.35; 95% CI [0.85–1.85], *p* < 0.001) and concerns regarding COVID-19 vaccine safety (ß= -4.09; 95% CI [-5.98; -2.21], *p* < 0.001) were associated with a negative attitude towards vaccination.

**Conclusion:**

Our findings reveal that COVID-19 vaccine acceptability among pregnant and breastfeeding women, amidst the pandemic was insufficient to meet community immunity. The identified reasons for vaccine reluctance, notably concerns about safety for both personal health and the health of their pregnancy or newborns, along with insufficient information about the vaccine, underscore the pressing need to address these factors to improve immunization rates.

**Supplementary Information:**

The online version contains supplementary material available at 10.1186/s13690-024-01267-8.

**Table:** 

Text box 1. Contributions to the literature
• Vaccine hesitancy was thought to be one of the largest threats to the human health.
• Positive attitudes regarding vaccinations were found among women who have preventative measure.
• The COVID-19 vaccine's acceptance level among pregnant and lactating women was insufficient.

## Background

According to the World Health Organization (WHO), pregnant women are classified among the most vulnerable groups of the population in the context of a pandemic or a disaster, having the highest risk of infection contraction and associated emergencies which would lead to increased maternal and fetal morbidity and mortality [1, 2]. The most important public health measure to fight against the COVID-19 pandemic and reduce its transmission is vaccination, since this disease is highly contagious and transmits rapidly [3, 4].

Vaccine hesitancy, defined as a delay in acceptance or refusal of vaccines despite the availability of vaccination services [5], is considered one of the biggest threats to human global health nowadays, even before COVID-19 pandemic. This concern became particularly evident with the emergence of COVID-19, especially with the lack of studies that show COVID-19 vaccine safety in pregnant and lactating women when the vaccines were first introduced [6]. The main barriers to vaccination during pregnancy are safety concerns, doubt about the need for vaccine or its effectiveness, lack of healthcare professional recommendation, limited knowledge about vaccines, access challenges, cost and conflicting advice [7]. According to a systematic review, contributors to Influenza and COVID-19 vaccine hesitancy were concerns about vaccine safety, lack of trust, lack of need for vaccination and cultural factors [8]. Based on a meta-analysis discussing factors affecting decision-making among pregnant women regarding vaccination, these factors include accessibility and convenience, awareness and perceptions regarding vaccination and its possible benefits and risks along with the social and emotional influence on vaccine use [9].

Additionally, anti-vaccination advocates campaigning against the vaccination concept in many countries rely most importantly on the speed and accelerated pace of COVID-19 vaccine development which increases public anxieties and affects vaccine acceptance widely [10]. Researchers are working on implementing large-scale vaccination campaigns and engaging health care professionals’ opinions directed to targeted audiences in order to increase awareness towards the importance of vaccination [10, 11].

In Lebanon, more than 5.6 million vaccine doses have been administered [12]. Regarding attitude towards vaccination in Lebanon, a study conducted on university students showed 87% acceptance towards vaccination which was associated with belief in vaccine safety [13]. The acceptance rate of COVID-19 vaccine was as high (86%) among Lebanese dentists associated with high vaccination knowledge and fear of COVID-19 [14] but was low as 21.4% in Lebanese adults in general [15]. A Lebanese study done among refugees and Lebanese nationals have found a low vaccine acceptance pre- vaccination (25.9% refugees vs. 23.1% Lebanese nationals) however, the percentages where higher post vaccination (Lebanese nationals (57.6%) versus refugees (32.9%)) [16]. In addition, according to a study conducted on the Lebanese population regarding the willingness to pay for COVID-19 vaccine, 68.2% were willing to take the vaccine when it is available [17].

Among the high number of COVID-19 vaccines developed since the start of the pandemic, none of the vaccines were studied in pregnant women. In addition, the FDA reviewed that the vaccine “did not have any adverse effects on female reproduction, fetal/embryonal development, or postnatal development” based on reproductive toxicity studies in rabbits [18]. Receiving the COVID-19 vaccine during pregnancy produces antibodies that can offer protection to the baby. Studies show that mRNA COVID-19 vaccines during pregnancy are effective. In addition, information about its safety before and during pregnancy are reassuring [19]. A systematic review and meta-analysis showed no safety concerns for COVID-19 vaccines that are currently administered during pregnancy [20]. Information from the animal developmental and reproductive toxicity (DART) studies of the Pfizer/BioNTech, Moderna, and Janssen vaccines indicates no safety issues. These studies show no adverse effects on female reproduction, fertility, embryonic or fetal development, or postnatal development, including miscarriage [21].

In this context, our study aimed to evaluate the attitude of pregnant and lactating women towards the COVID-19 vaccine in Lebanon and determine the factors that contributed to their acceptance or reluctance.

## Methods

### Study design and population

This was a quantitative, observational, cross-sectional study conducted in Lebanon between August and September 2021. The study included 207 pregnant and breastfeeding women from all Lebanese governorates (Beirut, Mount Lebanon, Bekaa, South and North). The eligibility criteria for participation were: [1] age 18 years old and above; [2] being pregnant or breastfeeding; [3] giving an oral informed consent. All eligible women were invited to participate in the study.

### Instrument

The questionnaire was developed based on a literature review. Some items were curated using validated instruments relevant to our study’s focus on attitude towards vaccination. To ensure alignment with our study context, modifications to selected items were made. Additionally, new items were crafted to highlight certain unexplored aspects in the literature [22–25]. It was developed on Google Forms in English and in Arabic, the native language in Lebanon. A pilot study, involving a convenient sample (*n* = 10) collected through the snowball technique, was conducted before the start of the main study. The sample comprised pregnant and breastfeeding women of various ages, professions, and educational qualifications to assess the face validity of the survey. Notably, no modifications to the questionnaire were required. The pilot study also validated the feasibility of the chosen sampling strategy, demonstrating its efficacy in capturing diverse perspectives. The main study retained the refined survey instrument and adopted the successful sampling approach identified during the pilot phase.

It included open and closed-ended questions and consisted of 5 sections. It was posted on different social media platforms and used in face-to-face interviews.

The first part included questions about maternal demographics and socioeconomic characteristics, including age, nationality, place of residence, educational level, participant’s and spouse’s employment status, income and insurance, presence of underlying medical chronic conditions along with medication history that are not related to pregnancy or breastfeeding and their current pregnancy/ breastfeeding status [25].

Questions regarding the presence of any risk factors for SARS-CoV-2 including chronic illnesses, smoking (including cigarettes and waterpipes), and physical activity (strength or aerobic training, swimming…) were asked for both pregnant and lactating women. The participants were assessed for the previous infection of COVID-19, along with complications or hospitalization history related to coronavirus infection [24]. Furthermore, previous vaccination against coronavirus was assessed including the type of vaccine used.

The second part was directed to pregnant women, including their current pregnancy status or trimester, number of gestations, conception method and previous miscarriages if present. In addition, the occurrence of any pregnancy complications like gestational diabetes, gestational hypertension and others, and previous intake of recommended vaccines during pregnancy was also addressed.

The third part was directed to breastfeeding women and included questions regarding the breastfed baby age, current breastfeeding complications (sore and cracked nipples, blocked milk duct…) and previous breastfeeding experience.

The fourth part included information regarding the attitude of pregnant and breastfeeding women towards COVID-19 pandemic. Preventive measures against coronavirus, reasons for considering stopping breastfeeding, belief about the effect of COVID-19 on pregnancy or infants, and awareness about the seriousness of COVID-19 pandemic were assessed.

The fifth and final part discussed the attitude of pregnant and lactating women towards COVID-19 vaccination, using a 5-point Likert scale varying from 1 (strongly disagree) to 5 (strongly agree). A scale was created by summing up eight questions, and the total score ranged from 9 to 39, with higher score indicating positive attitude towards vaccination against coronavirus. For example, the asked questions were, “I would get the COVID-19 vaccine during pregnancy or breastfeeding whenever it’s available, “I believe more studies are needed to ensure the safety and efficacy of COVID-19 vaccines during pregnancy/ breastfeeding”. In addition, questions regarding the reasons they would (or wouldn’t) get the vaccine were addressed to the participants [24, 25].

### Data collection

The data was collected using a questionnaire distributed either online or in person. The first method employed a snowball sampling technique, through an online web-based survey posted on social media platforms (Facebook and WhatsApp). The initial participants were asked to complete the survey and share the survey link with other pregnant and breastfeeding individuals in their social networks.

The second method was a paper survey distributed in gynecology and pediatric clinics that were chosen by convenience, based on their accessibility and willingness to participate in the study. The presence of the target population in these healthcare settings is of natural process given that pregnant and breastfeeding women frequently visit these clinics to seek healthcare monitoring during this period. After permission was granted from the site physicians and giving them clear explanations for the goals and importance of the study, the researcher invited patients waiting for their appointment in the outpatient clinics to participate. Each participant was approached individually, and they were ensured that their participation was completely anonymous, confidential and voluntary, and they were recruited after giving the informed consent. The successive recruitment process involved identifying potential participants among those present in the clinics during the data collection period. The interviewer was well trained in order to collect the data in an objective manner.

### Sample size calculation

For the sample size calculation, we used Epi Info™ software version 7.2.4.0.

According to previous studies showing the attitude of Lebanese adults in Lebanon towards COVID-19 vaccination, we used the proportion of positive attitude towards COVID-19 vaccination, which was 21.4% [15]. This proportion was crucial for calculating the minimum sample size, ensuring a precision level of 5% and a confidence interval of 95%. Accordingly, the minimal sample size is estimated to be 258. This choice of a 5% precision level reflects the standard practice in statistical analysis, allowing for a reliable estimation of the population parameter.

### Statistical analysis

A descriptive analysis was performed where categorical variables were expressed using numbers and percentages and quantitative measures using means ± standard deviations (SD).

The questions used to evaluate the attitude of pregnant and breastfeeding women towards the COVID-19 vaccine were adapted after a rigorous literature review, from previous studies conducted among the general population to develop an eight-item scale called Attitude scale. All items were graded on a 5-point Likert scale from 1 (strongly disagree) to 5 (strongly agree). The overall attitude score was calculated by adding the eight responses, with a higher score indicating a more positive attitude toward COVID-19 vaccine.

Principal component analysis was used to assess the construct validity of the Attitude Scale. Varimax rotation was applied since the extracted factors were significantly correlated without it. The model’s adequacy was ensured by calculating Kaiser–Meyer–Olkin’s measure of sampling adequacy and Bartlett’s test of sphericity. Factors with eigenvalues values greater than one were kept, and number of components to be extracted was determined using the scree plot method. In addition, items with loading over 0.4 were considered only. Moreover, the internal consistency of the Attitude Scale was assessed using Cronbach’s alpha.

The normality of the Attitude Scale was ensured using the histogram and the scale was used as a dependent variable.

A bivariate analysis was conducted using Student T-test and One-Way Anova to compare continuous variables with two or more levels, respectively. The differences between multiple group means were identified using Post Hoc test (Bonferroni). The Kruskall-Wallis test was applied whenever Levene’s Test (homogeneity of variances) was significant.

In the multivariable analysis, a linear regression was conducted using the forward method and taking the Attitude Scale as dependent variable and all the variables with a p-value < 0.004 in the bivariate analysis were included in the model. Bonferroni correction was used to calculate the p-value cut point (< 0.004) to include the most important variables in Linear regression, which in its turn will be used to estimate the association between baseline characteristics and vaccine acceptability. Then the linear regression was adjusted over different factors that consist of significant association with the scale.

For all statistical tests, a *p*-value less than 0.05 was considered significant and the confidence interval was 95%. The statistical analysis was performed using SPSS version 23 (IBM Corporation, Armonk, NY, USA).

## Results

### Participants’ characteristics

In total, 207 women participated in the study, out of which 132 were breastfeeding and 74 were pregnant and 1 pregnant and breastfeeding. One-third of the total population was recruited through the clinic-based method and two-thirds were recruited through the online survey. In addition, having one pediatrician clinic and two gynecologist clinics, the number of pregnant women recruited through the clinic-based sample was higher than that of lactating women.

Most responses were collected from Mount Lebanon (57%) and Beirut (14%). Around 45% of women were aged between 24 and 29 years. Participants’ sociodemographic characteristics are shown in Table 1.

**Table 1:** Socio-demographic characteristics of the 207 Lebanese women participated in the study during 2021 summer time

Variable		Frequency (percentage)	Mean ± Std. Deviation
**Demographic Characteristics:**			
Age	18–23	20 (9.7%)	29.14 ± 4.78
	24–29	94 (45.4%)	
	30–34	65 (31.4%)	
	35+	28 (13.5%)	
Nationality	Lebanese	189 (91.3%)	
	Non-Lebanese	18 (8.7%)	
Social status	Married	205 (99.0%)	
	Single	2 (1.0%)	
Place of residence	Mount Lebanon	118 (57.0%)	
	Beirut	29 (14%)	
	North	12 (5.8%)	
	South	21 (10.2%)	
	Bekaa	27 (13.0%)	
Educational level	None	7 (3.4%)	
	School Education	18 (8.6%)	
	Bachelor’s Degree	97 (46.9%)	
	Postgraduate Studies (Masters, PhD…)	85 (41.1%)	
**Employment Details:**			
Employment status of participant	Housewife	102 (49.3%)	
	Employed in other fields	82 (39.6%)	
	Employed in medical field	23 (11.1%)	
Employment status of your husband/ partner	Unemployed	12 (5.8%)	
	Employed in other fields	177 (85.5%)	
	Employed in medical field	16 (7.7%)	
Family monthly income	Low (< 3,000,000 LL − 8,999,999 LL)	102 (49.3%)	10,470,000 ± 6,786,000
	Intermediate: (9,000,000–17,999,999 LL)	83 (40.1%)	
	High (> 18,000,000 LL)	22 (10.6%)	

With regard to COVID-19, 38.6% (*n* = 80) have tested positive. Around 45% of women were vaccinated (*n* = 93) and the most administered vaccine was BioNTech, Pfizer (38.2%, *n* = 79).

The pregnant women were mostly in their third trimester (34.7%, *n* = 26), and most of the pregnant women didn’t take their recommended vaccines during pregnancy (85.3%, *n* = 64). In addition, a large percentage of pregnant women suffered from pregnancy health issues (46.7%, *n* = 35), mostly from iron deficiency anemia (22.7%, *n* = 19) and depression and anxiety (16%, *n* = 12).

Regarding the breastfed babies, most of them were above 6 months (46.4%, *n* = 48). More than 50% of breastfeeding women have experienced complications during breastfeeding (*n* = 69), where they suffered mostly from sore and cracked nipples (75.4%, *n* = 75), breast engorgement (47.8%, *n* = 33), followed by a blocked milk duct (21.7%, *n* = 15).

### Attitude towards covid-19

Regarding hygienic practices against COVID-19, pregnant and breastfeeding women followed different measures, most importantly, wearing face mask (94.7%, *n* = 196), avoiding crowded and enclosed spaces (79.7%, *n* = 165).

One hundred-twenty women (58%, *n* = 120) considered themselves at risk for COVID-19 because of their work environment. The bigger percentage of women agreed that the coronavirus infection will affect the development of an unborn child or newborn (37.2%, *n* = 77), and 36.7% (*n* = 76) were undecided. Furthermore, 72.5% (*n* = 150) of women believe that COVID-19 pandemic is as serious illness.

### Attitude towards covid-19 vaccination

While 72.5% of women believe that vaccines, in general, are safe, there were 36.7% who believe that COVID-19 vaccine will affect their health, pregnancy, and/or unborn or newborn child (Table 2). When the participants were asked how they decided to get the COVID-19 vaccine, it was because they believed that it is essential for them and their baby’s health (67.6%).

**Table 2:** Attitude of Lebanese pregnant and lactating women towards COVID-19 vaccination (*n* = 207)

Statement		Frequency (percentage)
In general, I think vaccines are safe.	Strongly disagree	7 (3.4%)
	Disagree	10 (4,8%)
	Undecided/Neutral	40 (19.3%)
	Agree	112 (54.1%)
	Strongly agree	38 (18.4%)
Do you think it is important to get a vaccine to protect the people from COVID-19?	Strongly disagree	4 (1.8%)
	Disagree	13 (6.3%)
	Undecided/Neutral	35 (16.9%)
	Agree	91 (44.0%)
	Strongly agree	64 (30.9%)
Have you or someone you know ever had a bad reaction to a vaccine?	Strongly disagree	24 (11.6%)
	Disagree	36 (17.4%)
	Undecided/Neutral	42 (20.3%)
	Agree	75 (36.2%)
	Strongly agree	30 (14.5%)
Do you trust the pharmaceutical companies that they develop safe and effective COVID-19 vaccines	Strongly disagree	13 (6.3%)
	Disagree	22 (10.6%)
	Undecided/Neutral	63 (30.4%)
	Agree	87 (42.0%)
	Strongly agree	22 (10.6%)
Do you trust the ministry of health that they ensure the safety packaging of COVID-19 vaccines?	Strongly disagree	25 (12.1%)
	Disagree	33 (15.9%)
	Undecided/Neutral	60 (29.0%)
	Agree	63 (30.4%)
	Strongly agree	26 (12.6%)
I have concerns related to possible side effects of COVID-19 vaccines.	Strongly disagree	18 (8.7%)
	Disagree	26 (12.6%)
	Undecided/Neutral	44 (21.3%)
	Agree	79 (38.2%)
	Strongly agree	40 (19.3%)
I believe that COVID-19 vaccine will affect my health and/or pregnancy and/or unborn/newborn child.	Strongly disagree	18 (8.7%)
	Disagree	42 (20.3%)
	Undecided/Neutral	71 (34.3%)
	Agree	35 (16.9%)
	Strongly agree	41 (19.8%)
I believe that more research is/ studies are needed to ensure the safety and efficacy of COVID-19 vaccines during pregnancy/ breastfeeding.	Strongly disagree	4 (1.9%)
	Disagree	12 (5.8%)
	Undecided/Neutral	13 (6.3%)
	Agree	96 (46.4%)
	Strongly agree	82 (39.6%)
Do you prefer some COVID-19 vaccines (such as those made in Europe or America) over others made in other world countries?	Strongly disagree	12 (5.8%)
	Disagree	21 (10.1%)
	Undecided/Neutral	47 (22.7%)
	Agree	66 (31.9%)
	Strongly agree	61 (29.5%)
Whenever the coronavirus vaccine is available, I would get the vaccine during pregnancy/ breastfeeding.	Strongly disagree	31 (15.0%)
	Disagree	27 (13.0%)
	Undecided/Neutral	42 (20.3%)
	Agree	55 (26.6%)
	Strongly agree	52 (25.1%)

### Reasons for covid-19 vaccine reluctance and acceptance

Participants were asked about the reasons they would take the vaccine or have taken it, as well as the reasons unvaccinated women are not intending to take the vaccine. The reasons for vaccine acceptance were mainly to protect themselves from getting sick with COVID-19 (81.4%) and to protect their baby (73.8%), while the reasons for vaccine reluctance among unvaccinated women (55.1%), were concerns about vaccine safety for their pregnancy or their baby (59.4%), and concerns about vaccine safety for themselves (39.1%).

### Attitude scale

One attitude scale towards COVID-19 vaccine was applied to the whole sample. The total attitude score was created by adding all Likert scale items related to attitude. The summation of the questions ranged from 9 to 39 and the median was 26. A higher score indicated a positive attitude towards vaccination.

The scale showed no over-correlation (*r* > 0.8), neither low loading factor (< 0.3) or low communalities (< 0.3). It was converged over a solution of one factor with an eigenvalue over 1, and accounting for a variance of 68.78%, KMO = 0.782 and significant Barlett sphericity test (*p* < 0.001), Cronbach’s alpha was 0.823 (Table 3).

**Table 3:** Factor Analysis of the attitude scale towards COVID-19 vaccine (Varimax rotated matrix)

Items	Factor 1	H2 communalities
In general, I think vaccines are safe	0.897	0.810
Do you trust the pharmaceutical companies that they develop safe and effective COVID-19 vaccines	0.871	0.767
Do you think it is important to get a vaccine to protect the people from COVID-	0.841	0.715
Do you trust the ministry of health that they ensure the safety packaging of COVID-19 vaccines	0.661	0.453
Whenever the coronavirus vaccine is available, I would get the vaccine during pregnancy/ breastfeeding.	0.787	0.667
I believe that more research is/ studies are needed to ensure the safety and efficacy of COVID-19 vaccines during pregnancy/ breastfeeding	0.716	0.522
I believe that COVID-19 vaccine will affect my health and/or pregnancy and/or unborn/newborn child/	0.858	0.788
I have concerns related to possible side effects of COVID-19 vaccines	0.882	0.788

### Factors associated with attitude towards covid-19 vaccination

Bivariate associations showed that pregnant and breastfeeding women with younger age under 34 have significantly more positive attitude towards COVID-19 vaccine, between 30 and 34 (25.8) vs. above 35 (21.86) (*p* = 0.028). Residents in South Lebanon (26.62) tended towards vaccine acceptance as compared with other Lebanese regions (North = 20.58) (*p* < 0.05).

In addition, participants who took the vaccine previously showed also a higher cumulative score on the attitude scale (34.71, *p* < 0.001), and compliance to preventive measures against coronavirus, was associated with a higher score on the attitude scale. However, women who believe that coronavirus infection will affect their unborn child/ newborn tended towards vaccine reluctance (*p* = 0.033).

Interestingly, belief in COVID-19 pandemic severity was shown to be associated with negative attitude towards vaccination (*p* = 0.039). In addition, women who did not experience a previous bad reaction to a vaccine had a higher score on the attitude scale (Disagree = 28.14, Neutral = 24.67, Agree = 23.77, Strongly agree = 25.27 *p* = 0.004).

Regarding the factors that affected women’s decision on taking the vaccine, the gynecologist recommendation (28.4, *p* < 0.001) and belief in the vaccine importance for one’s and baby’s health (29.34, *p* < 0.001) were associated with a positive attitude towards the vaccine.

Moreover, the reasons women have positive attitude towards the vaccine were mainly the protection of themselves (27.63), their baby (26.61), their family (26.97) and the community (27.33) against COVID-19 infection (*p* < 0.001).

On the other hand, concerns about vaccine safety for themselves (19.04) and their baby (21.34) (*p* < 0.001), in addition to concerns about vaccine efficacy (21.52, *p* = 0.007) were associated with negative attitude towards COVID-19 vaccine. Similarly, taking the vaccine without being convinced showed a negative association with the attitude scale (21.13, *p* = 0.009) (Table 4) (Table 5). (Table 6).

**Table 4:** Association between lifestyle and health conditions and attitude towards COVID-19 vaccination during summer 2021 among Lebanese pregnant and lactating women (*N* = 207)

Independent Variable		Total Attitude	
		***Mean + Std. Deviation***	***P-value***
Smoking status (cigarettes, e-cigarettes, waterpipes)	Current smoker (before and during pregnancy/breastfeeding)	24.32 + 6.74	0.859
	Ex-smoker (stopped before pregnancy/breastfeeding)	23.73 + 5.32	
	Non-smoker	24.95 + 5.90	
	Smoker but stopped during pregnancy/breastfeeding	25.04 + 5.99	
	**Total**	24.83 + 5.93	
Do you consider yourself physically active (cardio, tennis, gym…)?	No	24.55 + 6.20	0.310
	Yes	25.45 + 5.29	
Do you currently suffer from any chronic illness (not related to your pregnancy/breastfeeding)?	No	24.93 + 5.86	0.481
	Yes	24.04 + 6.51	
Do you suffer from Hypertension?	No	24.92 + 5.94	0.100
	Yes	20.00 + 2.31	
Do you suffer from Diabetes?	No	24.79 + 5.95	0.524
	Yes	27.00 + 5.20	
Do you take any chronic medications?	No	25.09 + 5.67	0.177
	Yes	23.00 + 7.39	
If yes, do you take Euthyrox as a chronic medication?	No	24.60 + 6.03	***0.010***
	Yes	27.50 + 3.72	
If yes, do you take PPI as a chronic medication?	No	24.84 + 5.99	0.277
	Yes	24.00 + 1.15	
Have you ever tested positive for COVID-19 infection?	No	24.35 + 6.88	0.106
	Yes	25.58 + 3.92	
Have you ever taken the COVID-19 vaccine?	No	28.46 + 5.49	***< 0.001***
	Yes	34.71 + 5.79	
Are you pregnant or breastfeeding?	I am currently breastfeeding	25.11 + 5.60	0.422
	I am currently pregnant	24.26 + 6.50	
	Both	30.00	
	**Total**	24.83 + 5.93	

A significant outcome is indicated by a *P* < 0.05. The bolded values are significant

**Table 5:** Association between compliance to preventive measures against COVID-19 and attitude towards COVID-19 vaccination during summer 2021 among Lebanese pregnant and lactating women (*N* = 207)

Independent Variable		Total Attitude	
		***Mean + Std. Deviation***	***P-value***
Do you wash your hands more often because of coronavirus?	No	22.71 + 5.01	***0.042***
	Yes	25.16 + 6.01	
Do you use alcohol-based hand sanitizer more often because of coronavirus?	No	23.55 + 5.79	0.177
	Yes	25.07 + 5.94	
Do you wear a face mask because of coronavirus?	No	25.82 + 3.87	0.570
	Yes	24.77 + 6.03	
Do you avoid public transport because of coronavirus?	No	24.86 + 5.76	0.960
	Yes	24.81 + 6.02	
Do you avoid crowded and/or enclosed spaces because of coronavirus?	No	25.88 + 5.65	0.197
	Yes	24.56 + 5.99	
Do you practice social distancing because of coronavirus?	No	25.80 + 5.55	0.183
	Yes	24.52 + 6.03	
Do you touch your face less because of coronavirus?	No	23.21 + 5.58	***< 0.001***
	Yes	26.25 + 5.88	
Do you shop for groceries less often because of coronavirus?	No	24.30 + 6.70	0.727
	Yes	24.98 + 4.90	
Do you cook at home more often because of coronavirus?	No	24.88 + 5.98	0.913
	Yes	24.79 + 5.92	
Do you purchase extra supplies or food because of coronavirus?	No	24.72 + 6.08	0.677
	Yes	25.11 + 5.57	
Do you consider yourself at risk for COVID-19? (because of your work, environment, etc.	No	25.26 + 6.11	0.367
	Yes	24.51 + 5.80	
I believe that a coronavirus infection during pregnancy/ breastfeeding will affect the development of an unborn child/ newborn.	Strongly disagree	28.11 + 7.32	***0.033***
	Disagree	26.08 + 5.21	
	Undecided/Neutral	24.57 + 4.87	
	Agree	24.41 + 5.86	
	Strongly agree	22.12 + 7.84	
	**Total**	24.83 + 5.93	

A significant outcome is indicated by a *P* < 0.05. The bolded values are significant

### Factors affecting attitude towards covid-19 vaccination

A multiple linear regression was conducted taking the COVID-19 vaccination attitude scale as a dependent variable. The results revealed an R-squared value of 0.558, indicating that approximately 55.8% of the variance in the attitude scale was accounted for by the included predictors. The adjusted R-squared, a measure that adjusts for the number of predictors in the model, was 0.545, reflecting a slightly lower but still substantial proportion of explained variance. These findings suggest that the model exhibits a moderate level of overall fit, capturing a noteworthy portion of the variability in the attitude scale.

Application of preventive measures against the pandemic like touching their face less (ß=2.25, *p* < 0.001) was significantly associated with positive attitude towards vaccine acceptance. In addition, the main motive of pregnant and breastfeeding women for getting the vaccine was to protect themselves against COVID-19 infection (ß=4.22; *p* < 0.001). Similarly, the belief that the vaccine is essential for them, and their baby had a positive influence on their attitude towards the vaccine (ß=3.49, *p* < 0.001). While experiencing a bad reaction to a vaccine and having concerns about vaccine safety for themselves were significantly associated with negative attitude (ß= -1.35, ß= -4.09 *p* < 0.001).

When assessing sociodemographic characteristics, no statistically significant differences were found between age, nationality, marital status, education, employment, smoking, chronic diseases, type of conception, pregnancy trimester at survey and pregnancy complications during current pregnancy and others, and acceptability towards COVID-19 vaccine.

**Table 6:** Linear repression analysis using the forward method and taking the attitude towards COVID-19 vaccination as a dependent variable among Lebanese pregnant and lactating women (*N* = 207)

Statement	Unstandardized B	Standardized Beta	P-value	95.0% Confidence Interval for B
Do you touch your face less because of coronavirus? (ref: did not touch your face)	2.25	0.16	***< 0.001***	[1.02; 3.48]
Have you or someone you know ever had a bad reaction to a vaccine? (ref: strongly disagree)	-1.35	-0.26	***< 0.001***	[0.85–1.85]
Do you prefer some COVID-19 vaccines (such as those made in Europe or America) over others made in other world countries? (ref: strongly disagree)	1.23	0.22	***< 0.001***	[0.69–1.77]
If you already took the vaccine, you decided to take it because you believe it is essential for you and your baby’s health. (ref: not essential)	3.49	0.26	***< 0.001***	[2.01–4.98]
You would take/took the COVID-19 vaccine to protect yourself from getting sick with COVID-19. (ref: No)	4.22	0.33	***< 0.001***	[2.83; 5.61]
You wouldn’t take the COVID-19 vaccine because you have concerns about vaccine safety for yourself. (ref: No)	-4.09	-0.21	***< 0.001***	[-5.98; -2.21]

A significant outcome is indicated by a *P* < 0.05. Numbers in bold and italic indicate significant *p*-values

## Discussion

Our main findings showed that the acceptance of the COVID-19 vaccine among pregnant and breastfeeding women was 51.7%, which appears to be lower than the acceptance rate observed in the general Lebanese population during the same period, which was reported at 63.4% [26] and 57.6% [16] This percentage aligns favorably compared to certain international studies, surpassing rates found in two US studies on pregnant women (41%,44.3%) [24, 27], the Middle Eastern population (36.8%) [28], the general Lebanese population (21.4%) [15], Switzerland (35.7%) [29], and approximately similar to a survey conducted in 16 countries (52%) [6]. On the other hand, it falls short when compared with global survey data from 19 countries was (71.5%) [10], and studies conducted in China (91.3%) [30], Saudi Arabia (64.7%) [31], United States (57%) [32] and Southwest Ethiopia (70.9%) [33]. This nuanced comparison underscores the variability in vaccine acceptance rates across diverse populations and geographical locations.

Our study showed that vaccination acceptance was associated with positive attitude towards preventive measures (ß=2.25), preference for vaccine made in Europe and America (ß=1.23) and a belief in the importance of vaccination for themselves and their babies (ß=3.49). While negative attitude towards vaccination was associated with experiencing a previous bad reaction to a vaccine (ß= -1.35) and concerns regarding COVID-19 vaccine safety (ß= -4.09).

It is evident that pregnant women in their first trimester with low parity and low-risk gestational profile women are hesitant to receive the vaccination due to fears from potential teratogenic effects and concerns regarding the safety of the fetus potentially resulting in complications during pregnancy [33, 34]. The general COVID-19 vaccine safety was a primary reason for delaying COVID-19 vaccination, with 85.44% expressing concern about the impact on their own body and the potential effects on the unborn child [36].

In exploring the multifaceted landscape of COVID-19 vaccine acceptance among pregnant and breastfeeding women in Lebanon, it is important to delve into the intricate web of cultural, political, and historical factors that shape these attitudes. Religion-related convictions have an important role in reluctance towards vaccination, which was shown to be connected to a belief in divine protection and healing among different religions [32, 37]. Since Lebanon has a diverse cultural tapestry, characterized by a mosaic of religious beliefs and practices. This can significantly influence health-related decision-making, and the impact of religious leaders and institutions on vaccine acceptance warrants careful examination. Moreover, the political climate in Lebanon, marked by periods of instability and governance challenges, can contribute to a broader sense of distrust among the population. Interestingly, countries with the highest acceptance rates were mostly Asian nations where there is high trust in central governments and in middle-income countries [10]. And this might explain the cause of which our study showed a lower vaccine acceptance rate where there is distrust in central governments. This finding is also supported by studies that indicate the main barrier for vaccine acceptance is mistrust in governmental policies [31, 38]. Studies show that when trust levels are at their lowest, around 20%, less than half of the population is likely to accept vaccines. Conversely, when trust levels are at their highest, approaching 100%, the acceptance rate for a no-cost vaccine rises to nearly 80% [39]. Distrust in science and pharmaceutical companies significantly is also identified as a significant contributor for vaccine hesitancy [40]. Moreover, political factors, including conflicting messages regarding COVID-19 from both community members and political leaders, coupled with uncertainties about the existence of the virus and the efficacy of the vaccine and holding conservative political views across all metropolitan areas are associated with higher vaccine hesitancy [27, 35, 41, 42]. This should evoke the Lebanese government to raise awareness about the importance of vaccination against COVID-19 along with equal distribution of vaccines among the population and increase the trust of the population in governmental health practices.

Furthermore, vaccine hesitancy has been significantly influenced by historical incidents that eroded public trust, with one notable example being the paper authored by Andrew Wakefield, where he suggested a connection between the MMR (measles, mumps, and rubella) vaccine and autism. Despite the retraction of the paper and the identification of serious flaws in Wakefield’s research, the lasting impact on vaccine compliance has been profound [43]. And this issue traced back to Edward Jenner’s smallpox vaccination in the 1800s, faced diverse objections in England and the US, where anti-vaccination leagues and societies were formed in response to mandatory laws of vaccination against smallpox. Later, the DTP vaccine controversy also demonstrated enduring challenges despite scientific evidence refuting adverse effects [44]. Such historical incidents contribute to the persistence of vaccine hesitancy, highlighting the importance of transparent communication, robust scientific scrutiny, and ongoing efforts to rebuild trust in vaccination programs.

A study conducted in Lebanon in 2018 showed low awareness of Lebanese gynecologists and obstetrics regarding the CDC/ACIP immunization schedule for women in general, where only 62.3% recommended vaccination to pregnant women and only 25.9% recommended the Tdap vaccine during the third trimester [45]. These results were consistent with the low vaccination percentage of recommended vaccines during pregnancy in our study (14.7%) and explain partially one of the reasons that might affect the attitude of women in Lebanon and especially pregnant and breastfeeding women towards vaccination. This finding is supported by another study conducted in Lebanon that showed a high knowledge and perception of pregnant women about COVID-19 illness, which suggests strong responsibility towards their fetuses and the illness, but better communication and planning with their physicians help with reaching the goal in vaccination to attain better health care and control the pandemic [46].

The investigation into factors influencing attitudes toward COVID-19 vaccination reveals a complex interplay of considerations. According to our findings, the strongest factors associated with positive attitude towards COVID-19 vaccine were compliance with public health measures such as touching the face less, belief in the importance, efficacy and safety of the vaccine as well as worry about getting sick with COVID-19. Compliance with recommended public health measures, such as reduced face-touching and adherence to social distancing, signifies a heightened awareness of the gravity of the pandemic. This proactive commitment to preventive behaviors likely contributes to a positive attitude by demonstrating an individual’s dedication to minimizing the risk of infection. And this is demonstrated in a cross-sectional survey from 16 countries including United States, Brazil, India, Russia, Spain, Australia and others, compliance regarding public health measures, including face masks and social distancing was above 75% in these countries [6].

Furthermore, the alignment of beliefs in the importance, efficacy, and safety of the vaccine, were also supported by the findings of this cross-sectional survey across 16 countries. A significant proportion of both pregnant and non-pregnant women in the study from these countries were confident about any newly approved vaccine would be safe, with no harmful adverse effects (53%), and believed it would be effective and protective against the pandemic (60.4%) [6]. This underscores the global significance of public confidence in the vaccine’s safety and effectiveness. The observed positive attitude may be driven by a recognition of the vaccine’s crucial role in preventing illness. Heightened worry about contracting COVID-19 further underscores the perceived threat and urgency for protection, reinforcing a positive attitude towards vaccination.

Regardless of the vaccine’s efficacy and safety, origin and brand of the vaccine were important drivers of vaccine acceptance. In a Chinese study, the probability of accepting BioNTech was 31% higher than that of Sinovac, whereas AstraZeneca exhibited a decreased likelihood by 11% [47]. According to our findings, a preference of vaccines made in Europe and America is evident which could explain the higher level of trust in certain countries over others among the population in Lebanon. This preference affects people’s willingness to take the vaccine depending on the type of vaccine that is available. A similar trend is observed in a Jordanian study, where one-third of the participants perceived COVID-19 vaccines manufactured in America and Europe to be safer than those made in other countries [25]. This regional preference may be influenced by historical, cultural, and geopolitical elements that shape perceptions of vaccine quality and reliability. Trust in the regulatory processes of these regions, known for stringent standards and rigorous testing, could foster a positive perception of the vaccines produced therein. Additionally, the historical success and reputation of Western pharmaceutical companies may contribute to this confidence. Cultural familiarity and exposure to vaccines developed in these regions through extensive global distribution networks may also play a role in shaping preferences [48]. Geopolitical stability and robust healthcare systems in these areas could further enhance trust [49, 50].

According to a cross-sectional study in Turkey done on 300 pregnant women, decreased confidence about vaccine efficacy and the social influence on perceptions towards the vaccine, and lastly the lack of safety data and studies in pregnant women were main barriers affecting vaccine acceptance [51]. The reasons for this perception were mainly worrying about their neonate health and the rush in vaccine development which took much less time to be developed than previous vaccines [52]. In addition, vaccine safety appeared to be a main factor associated with vaccine acceptance [32, 38]. And these results were consistent with our study results.

On the contrary, the experience of a bad reaction to a vaccine by the pregnant or breastfeeding women themselves or their acquaintances was associated with negative attitude to the COVID-19 which is consistent with other studies that showed the effect of concerns and fear from side effects on vaccine hesitancy [53–55].

Similarly, our study showed that concerns about COVID-19 vaccine safety for pregnant and breastfeeding women is a strong factor related to vaccine hesitancy, which was consistent with another study in the US [24] and several studies identifying worry about COVID-19 vaccine side effects that might affect their newborn or fetus, in addition to concerns regarding its effectiveness [29, 56]. Women are reluctant due to the lack of sufficient research during pregnancy and breastfeeding to ensure the vaccine’s safety and efficacy in this population. And that explanation is supported by the large percentage of women in our study who believed that more studies on the vaccine are needed during pregnancy and breastfeeding (86%).

Some recent studies showed that the vaccine was well tolerated in pregnant and lactating women with mild short-term side effects including pain at the injection site and fatigue. This might have a great influence on the significant factors affecting the choice to get vaccinated, providing more data on these populations, in consequence, increasing the trust in the vaccine built on scientific reviews [57–60].

In conclusion, addressing identified barriers and concerns among pregnant and lactating women is crucial for improving vaccine acceptance within this population in the Lebanese healthcare context. Tailoring communication strategies to highlight the rigorous regulatory standards and historical success of vaccines from various regions, irrespective of their origin, could enhance public confidence. Interventions should include targeted educational campaigns to provide accurate information about vaccine safety, engaging healthcare professionals in open conversations, and acknowledging the unique concerns of pregnant and lactating women. Implementing accessible vaccination clinics, offering personalized information based on individuals’ experiences, and organizing community engagement programs led by local leaders and influencers can further alleviate apprehensions. Leveraging social media and technology to disseminate accurate information is key, along with fostering a global dialogue through interactive public health campaigns to promote broader acceptance and participation in public health initiatives.

### Strengths and study limitations

It is the first study that assesses the attitude towards COVID-19 vaccination in Lebanon in a vulnerable population which is pregnant and breastfeeding women. In addition, this target population is challenging, especially in the vaccination process since they’re not included in clinical trials, yet we gathered new important information about vaccination in Lebanon. Our study is instrumental in filling this void, offering valuable insights that can inform targeted public health policies in Lebanon. By delving into the attitudes of pregnant and breastfeeding women, we contribute essential data for designing and implementing effective vaccination strategies that prioritize the safety of both mothers and infants. Moreover, it provides healthcare providers and policymakers with crucial information to tailor communication strategies and address concerns. The study has also some limitations: it is a cross-sectional study and there is no temporal sequence, hence there is no causal relationship between attitude towards vaccination and associated variables. Additionally, a shortfall in our target sample size of 258 to 207 was encountered, primarily due to practical constraints caused by certain economic circumstances in Lebanon and COVID-19 emergence during the data collection period. This discrepancy of 51 samples warrants consideration, as it may impact the study’s statistical power, potentially influencing the reported significance of our findings.

We used two data collection methods without doing sensitivity analysis which generates another limitation to the study. The convenience sampling approach employed may not fully capture the sociodemographic diversity of the entire Lebanese population. Since clinics were concentrated in a specific area (Mount Lebanon), and our sample displayed a higher representation of individuals in suburban areas, these lead to discrepancies in socioeconomic status and geographical representation, influencing the external validity and potentially limiting the generalizability of our findings. Weighing technique to adjust the results to accurately represent the target population is challenging due to the absence of recent data.

While the dual-mode data collection approach allowed for a broader inclusion of participants, we acknowledge the possibility of introducing biases. Access bias, information, response and geographical biases were associated with each method. Snowball technique introduces selection bias which might lead to gathering pregnant and breastfeeding women with higher educational level and better medical knowledge. Face-to-face data collection method also cause selection bias which includes participants mainly in Mount-Lebanon, and with higher economic situation able to afford a doctor’s visit. In addition, it might cause interviewer inaccuracy and therefore interviewer bias.

Non-response could occur during the snowball sampling process if participants choose not to share the survey link or if convenience-sampled individuals decline participation in face-to-face interviews due to various reasons, such as time constraints or personal factors. While adjustments for non-response or incomplete surveys were not explicitly integrated into the initial sample size calculation, we recognize the potential impact of non-response on the study’s outcomes. Efforts were made to minimize non-response by implementing clear instructions, reminders, and user-friendly survey design. However, it’s important to acknowledge that non-response or incomplete surveys may still introduce biases to some extent. Moreover, while we employed multivariate analysis to control for known confounding variables, the absence of a sensitivity analysis is acknowledged as another limitation to the study. Consequently, the residual confounding bias is possible, as certain unmeasured factors that might be associated with attitude towards COVID-19 vaccination were not assessed.

## Conclusion

Our findings reveal that COVID-19 vaccine acceptability among pregnant and breastfeeding women, amidst the pandemic was insufficient to meet community immunity. The identified reasons for vaccine reluctance, notably concerns about safety for both personal health and the health of their pregnancy or newborns, along with insufficient information about the vaccine, underscore the pressing need to address these factors to improve immunization rates.

As we conclude this study, we must shift our focus towards future pandemics and incorporate the lessons learned from COVID-19. Recognizing the importance of ongoing research, future studies should deepen our understanding of cultural, historical, and societal factors influencing vaccine attitudes in this population. Longitudinal studies tracking attitude changes and assessing the impact of public health campaigns will provide insights into the sustainability of interventions. Proactive strategies, including targeted awareness campaigns, equitable access measures, and trust-building initiatives are vital for enhancing vaccine acceptance and protecting populations during future health crises.

### Electronic supplementary material

Below is the link to the electronic supplementary material.

Supplementary Material 1

## Data Availability

The data used and/or analyzed in this study is available and will be provided by the corresponding author upon request.

## Abbreviations

WHO: World Health Organization
FDA: Food and Drug Administration
CDC: Centers for Disease Control and Prevention
SD: standard deviations
SPSS: Statistical Package for the Social Sciences
KMO: Kaiser–Meyer–Olkin
US: United States
ACIP: Advisory Committee on Immunization Practices; LL:Lebanese Lira
