# Supplementary material for: Estimating COVID-19 vaccine acceptance in pregnant and lactating women: a cross-sectional study in Lebanon
Source: Arch Public Health. 2024 Mar 18;82:38. doi: 10.1186/s13690-024-01267-8 (PMC10949662; doi:10.1186/s13690-024-01267-8)
Supplement: Supplementary file 1 — Supplementary Material 1 [file 13690_2024_1267_MOESM1_ESM.docx]

**Questionnaire:**

**PART 1: SOCIO-DEMOGRAPHIC CHARACTERISTICS / الخصائص الاجتماعية والديموغرافية**

1. Age (in years) / العمر (بالسنوات)

……………………………………………

1. Nationality/الجنسية :
2. Lebanese /لبنانية
3. Non-Lebanese/غير لبنانية
4. Place of residence/ محل الأقامة :
5. Mount Lebanon/ جبل لبنان
6. Beirut/ بيروت
7. North/ الشمال
8. South / الجنوب
9. Bekaa/ البقاع
10. Do you have kids other than your current pregnancy/ breastfed baby? هل لديك أولاد غير حملك الحالي أو ولدك الرضيع؟
11. Yes نعم
12. No كلا
13. If yes, do your kid(s) go to school, summer school or kindergarten? / إذا كانت الإجابة نعم ، فهل يذهب طفلك/أطفالك إلى المدرسة أو المدرسة الصيفية أو روضة الأطفال؟
14. Yes نعم
15. No كلا
16. Educational levelالمستوى العلمي :
17. School education المرحلة الاساسية او الثانوية
18. Bachelor شهادة البكالوريوس
19. Postgraduate studies (Masters, PhD…) دراسات عليا
20. None لا شيْ
21. Your employment status/ عملك :
22. Employed in medical field موظفة في قطاع الصحة
23. Employed in other fields موظفة في قطاعات أخرى
24. Housewife/ Unemployed ربة منزل/عاطلة عن العمل
25. Are you in frequent contact with people because of your work? / هل أنت في تواصل دائم مع الناس بسبب عملك؟
26. Always دائمًا
27. Often غالبًا
28. Sometimes أحيانًا
29. Rarely نادرًا
30. Never أبدًا
31. Are you directly caring for or in contact with COVID-19 patient(s)? / هل تهتمين بشكل مباشر أو على اتصال بمريض (مرضى) كوفيد-19؟
32. Yes نعم
33. No كلا
34. Your social status / الحالة الإجتماعية
    1. Married / ﻣﺗزوﺟﺔ
    2. Divorced / ﻣطﻠﻘﺔ
    3. Widowed / أرﻣﻠﺔ
    4. Other / غيره……………………
35. Employment status of your husband/ partner عمل الزوج/ الشريك :
36. Employed in medical field/ موظف في قطاع الصحة
37. Employed in other fields/ موظف في قطاعات أخرى
38. Unemployed/ عاطل عن العمل
39. Family monthly income الدخل الشهري للأسرة :
40. < 3,000,000 LL/ ل.ل
41. 3,000,000 – 5,999,999 LL/ ل.ل
42. 6,000,000 – 8,999,999 LL/ل.ل
43. 9,000,000 – 11,999,999 LL/ل.ل
44. 12,000,000 – 14,999,999 LL/ل.ل
45. 15,000,000 – 17,999,999 LL/ل.ل
46. >18,000,000 LL/ل.ل
47. Do you or your spouse/ partner have health insurance? /هل انت أو زوجك/شريكك لديك(ه) تأمين صحي؟
48. Yes, we have governmental/military health insurance / نعم لدينا تأمين صحي حكومي أو خدمات طبية
49. Yes, we have private health insurance/ تأمين صحي خاص
50. No, we don’t have any health insurance/ بدون تأمين صحي
51. Smoking status (cigarettes, e-cigarettes, shisha)/التدخين (سجائر، سجائر الكترونية، ارجيلة)
52. Current smoker (before and during pregnancy/breastfeeding)/ مدخنة حالية (خلال الحمل/الرضاعة)
53. Smoker but stopped during pregnancy/breastfeeding/ مدخنة لكن توقفت عن التدخين خلال الحمل/ الرضاعة
54. Ex-smoker (stopped before pregnancy/breastfeeding)/ مدخنة سابقة(توقفت قبل الحمل/الرضاعة)
55. Non smoker / غير مدخنة
56. Do you consider yourself physically active (cardio, tennis, gym...)? / هل تعتبرين نفسك نشيطة بدنيا (كارديو/تنس/نادي رياضي) ؟
57. Yes/ نعم
58. No/ كلا
59. Do you currently suffer from any of these chronic illnesses (not related to your pregnancy or breastfeeding)? (غير متعلقة بحملك/الرضاعة الطبيعية) هل تعانين حاليا أي من هذه المشاكل صحية
60. Heart Disease/ أمراض قلبية
61. Cancer/ سرطان
62. Chronic Lung Disease/ مرض الرئة المزمن
63. Stroke/سكتة دماغية
64. Hypertension/ضغط
65. Diabetes/سكري
66. Kidney Disease/مرض كلوي
67. None/لا يوجد
68. Other/ غيره …………………………………………………………………
69. Do you take any chronic medications? /هل تأخذين أي أدوية مزمنة
70. Yes/ نعم
71. No/ كلا
72. If yes, specify/ إذا نعم حدد……………………………………………………….
73. Have you ever tested positive for COVID-19 infection? / هل أثبت إصابتك بجائحة الكورونا من خلال الفحص المخبري؟
74. Yes/ نعم
75. No/ كلا
76. If yes, were you hospitalized? / إذا نعم هل دخلتي المستشفى؟
77. Yes/ نعم
78. No/ كلا
79. If you were infected, did you have any complications from your COVID-19 infection? / إذا أصبت سابقًا، هل عانيت من أي مضاعفات من إصابتك بفيروس الكورونا؟
80. Yes/ نعم
81. No/ كلا
82. Have you ever take the COVID-19 vaccine? /هل سبق و أخذت لقاح الكورونا؟
83. Yes/نعم
84. No/ كلا
85. If yes, which vaccine did you take? /إذا نعم أي نوع لقاح أخذت؟
86. BioNTech, Pfizer vaccine
87. Oxford, AstraZeneca vaccine
88. Sinopharm vaccine
89. Sputnik V vaccine
90. Are you pregnant or breastfeeding? /هل أنت حامل أو مرضعة؟
91. I am currently pregnant/أنا حامل
92. I am currently breastfeeding/أنني أرضّع
93. Both/ كلاهما

**PART 2: PREGNANCY/ الجزء الثاني: الحمل**

1. In which trimester are you? /في أي ثلث من الحمل أنت حالياً؟
2. First trimester/الثلث الأول
3. Second trimester/الثلث الثاني
4. Third trimester/الثلث الثالث أو الأخير
5. Have you been pregnant before? /هل حملت سابقاً؟
6. Yes/نعم
7. No/كلا
8. How many kids do you have in total (excluding your current pregnancy? / كم ولد لديك (باستثناء حملك الحالي)؟

…………………………………………………………

1. Have you had any previous miscarriage? /هل تعرضت لأي حالة إجهاض سابقاً
2. Yes/نعم
3. No/كلا
4. Do you suffer from any pregnancy health issues? /هل تعانين من أي مشاكل صحية متعلقة بالحمل
5. Yes/نعم
6. No/كلا
7. If yes, what do you suffer from? /إذا نعم ما الذي تعانين منه تحديداً؟
8. Gestational diabetes/ السكري الحملي
9. Gestational hypertension/ ارتفاع ضغط الدم الحملي
10. Iron deficiency anemia/ فقر الدم الناجم عن نقص الحديد
11. Depression and anxiety/ الإكتئاب والقلق
12. Infections/ الإلتهابات
13. Others/غيره………………………………………………………………………
14. Conception method/ طريقة الحمل
15. Spontaneous/ تلقائي
16. IVF/تلقيح إصطناعي/تلقيح أنابيب
17. Have you ever taken any recommended vaccines during pregnancy? (DTPa and/or influenza…) /هل سبق وأخذت أي لقاح موصى به خلال الحمل؟ (الإنفلونزا/ اللقاح الثلاثي المحسن)
18. Yes/نعم
19. No/كلا

**PART 3:** **BREASTFEEDING / الرضاعة الطبيعية**

If you are not breastfeeding, skip this section /

إذا كنت لا ترضعين طفلك ، فتجاوزي هذا القسم

1. How many kids do you have in total (including your breastfed baby)? / كم ولد لديك (متضمنًا طفلك الذي يرضع)؟

…………………………………………………………

1. Please indicate the age of the baby that is currently breastfed/ رجاء تحديد عمر الرضيع
   1. < 6 weeks/ أقل من 6 أسابيع
   2. Between 6 weeks and 6 months/ بين 6 أسابيع و6 أشهر
   3. > 6 months/أكثر من 6 أشهر
2. Have you ever experienced breastfeeding before? /هل مررت بتجربة الرضاعة سابقاً

Yes/نعم

No/كلا

1. Have you ever experienced any complications during breastfeeding? /هل سبق وحصل معك أي مضاعفات خلال الرضاعة؟
2. Yes/نعم
3. No/كلا
4. If yes, which complications did you suffer from/إذا نعم، من أي مضاعفات عانيت؟
5. Sore or cracked nipples/ألم وتشقق الحلمات
6. Not enough breast milk/ عدم توفر كمية الحليب الكافية
7. Breast engorgement/ إحتقان الثدي
8. Baby is not being able to drink milk properly/عدم إستطاعة الرضيع أن يرضع جيداً
9. Blocked milk duct/إنسداد قناة الحليب
10. Mastitis/إلتهاب الثدي
11. Other/غيره……………………………………………………………………………
12. Have you ever considered stopping breastfeeding during the pandemic? /هل فكرتي أن توقفي رضاعة طفلك خلال جائحة الكورونا؟
13. Yes/نعم
14. No/كلا

**PART 4: ATTITUDE TOWARDS COVID-19/ COVID-19الموقف تجاه**

1. Which of the following steps, if any, have you taken because of coronavirus? Select all that apply / أي من الخطوات التالية ، إن وجدت ، اتخذتها بسبب فيروس كورونا؟ اختري كل ما ينطبق
2. Washing hands more often / غسل اليدين في كثير من الأحيان
3. Using alcohol-based hand sanitizer more often / استخدام معقم اليدين الذي يحتوي على الكحول في كثير من الأحيان
4. Wearing a face mask / لبس قناع للوجه
5. Avoiding public transport / تجنب المواصلات العامة
6. Avoiding crowded and/or enclosed spaces / تجنب الأماكن المزدحمة و / أو المغلقة
7. Practice social distancing / ممارسة التباعد الاجتماعي
8. Touching your face less/ تقليل لمس وجهك
9. Shopping for groceries less often/ التسوق لشراء البقالة أقل من قبل
10. Cooking at home more often / الطبخ في المنزل أكثر من قبل
11. Purchasing extra supplies or food / شراء لوازم أو طعام إضافي
12. None/ لا شيء
13. Do you consider yourself at risk for COVID-19? (because of your work, environment, etc.) / هل تعتبرين نفسك معرضةً لخطر الإصابة بـكوفيد-19؟ (بسبب عملك ، بيئتك، إلخ.)
14. Yes/ نعم
15. No/ كلا
16. For breastfeeding women: If you considered stopping breastfeeding during the pandemic, what is/are the reason(s)? /إلى المرأة المرضعة: إذا فكرتي وقف إرضاع طفلك خلال هذه الجائحة، ما هو/هي السبب/الأسباب؟
17. I don’t/didn’t want to infect my baby while breastfeeding/لم/لا أرغب في إصابة طفلي أثناء الرضاعة الطبيعية
18. I felt too sick to breastfeed/شعرت بمرض شديد لدرجة أنني لا أستطيع الرضاعة الطبيعية
19. A healthcare professional advised me to stop breastfeeding/ نصحني أخصائي رعاية صحية بالتوقف عن الرضاعة الطبيعية
20. A family member / friend advised me to stop breastfeeding/ نصحني أحد أفراد العائلة أو صديق بالتوقف عن الرضاعة الطبيعية
21. I did not receive or find a reassuring answer to my questions about coronavirus and breastfeeding/ لم أتلقى أو أجد إجابات مطمئنة لأسئلتي حول فيروس كورونا والرضاعة الطبيعية
22. I was infected in COVID-19 and I use(d) a medicine that is incompatible with breastfeeding/ لقد أصبت بكوفيد-19 وأستخدم/ استخدمت دواء لا يتوافق مع الرضاعة الطبيعية
23. Due to concerns for the virus, I did not produce enough milk anymore/ بسبب مخاوف من الفيروس، لم أعد أنتج ما يكفي من الحليب
24. Due to the coronavirus, I had to work (more) and could no longer combine it with breastfeeding/ بسبب فيروس كورونا، اضطررت الى العمل (أكثر) ولم يعد بإمكاني التنسيق بينه وبين الرضاعة الطبيعية
25. Due to the coronavirus, I did not receive sufficient support from a healthcare professional wfith regard to breastfeeding and/or breastfeeding related issues/ بسبب فيروس كورونا، لم أتلق دعما كافيا من أخصائي الرعاية الصحية فيما يتعلق يالرضاعة الطبيعية و/ أو القضايا المتعلقة بالرضاعة الطبيعية

|  | Strongly Agree/ أوافق بشدة | Agree/ أوافق | Undecided/ Neutral/غير محدد/ محايد | Disagree/ لا أوافق | Strongly Disagree/ لا أوافق بشدة |
| --- | --- | --- | --- | --- | --- |
| 1. I believe that a coronavirus infection during pregnancy/ breastfeeding will affect the development of an unborn child/ newborn/ أعتقد أن الإصابة بفيروس الكورونا أثناء الحمل/ الرضاعة الطبيعية ستؤثر على نمو الجنين/ الطفل |  |  |  |  |  |
| 1. I believe that the COVID-19 pandemic is a serious illness/ أعتقد أن جائحة كوفيد-19 هو مرض خطير |  |  |  |  |  |

**PART 5: ATTITUDE TOWARDS COVID-19 VACCINATION / الموقف تجاه التطعيم ضد كوفيد-19**

|  | Strongly Agree/ أوافق بشدة | Agree/ أوافق | Undecided/ Neutral/غير محدد/ محايد | Disagree/ لا أوافق | Strongly Disagree/ لا أوافق بشدة |
| --- | --- | --- | --- | --- | --- |
| 1. In general, I think vaccines are safe/ بشكل عام، أعتقد أن اللقاحات آمنة |  |  |  |  |  |
| 1. Do you think it is important to get a vaccine to protect the people from COVID-19? / هل تعتقدين أنه من المهم الحصول على لقاح لحماية الناس من كوفيد-19؟ |  |  |  |  |  |
| 1. Have you or someone you have known ever had a bad reaction to a COVID-19 vaccine? /هل أصبت انت او اي شخص تعرفينه بمضاعفات سلبية جراء اللقاح؟ |  |  |  |  |  |
| 1. Do you trust the pharmaceutical companies to develop safe and effective COVID-19 vaccines? / هل تثقين في شركات الأدوية أنها تطور لقاحات كوفيد-19 آمنة وفعالة؟ |  |  |  |  |  |
| 1. Do you trust the ministry of health to ensure the safety packaging of COVID-19 vaccines? /هل تثقين بوزارة الصحة أنها تضمن سلامة عبوات لقاحات كوفيد-19؟ |  |  |  |  |  |
| 1. I have concerns related to possible side effects of COVID-19 vaccines/ لدي مخاوف تتعلق بالآثار الجانبية المحتملة للقاحات كوفيد-19 |  |  |  |  |  |
| 1. I believe that the COVID-19 vaccine will affect my health and/or pregnancy and/or unborn/newborn child/ أعتقد أن لقاح كوفيد-19 سيؤثر على صحتي و/أو حملي و/أو الجنين/ حديث الولادة |  |  |  |  |  |
| 1. I believe that more research/studies are needed to ensure the safety and efficacy of COVID-19 vaccines during pregnancy/ breastfeeding/أعتقد أن هناك حاجة إلى مزيد من البحوث/ الدراسات لضمان سلامة وفعالية لقاحات كوفيد-19 أثناء الحمل/ الرضاعة الطبيعيةCOVID-19 vaccines during pregnancy/ breastfeeding |  |  |  |  |  |
| 1. Do you prefer some COVID-19 vaccines (such as those made in Europe or America) over others made in other world countries? /هل تفضل بعض لقاحات كوفيد-19 (مثل تلك المصنوعة في أوروبا و أمريكا) على تلك المصنوعة في دول العالم الأخرى؟ |  |  |  |  |  |
| 1. Whenever the coronavirus vaccine is available, I will get the vaccine during pregnancy/ breastfeeding/ عندما يتوفر لقاح فيروس كورونا، سأحصل على اللقاح أثناء الحمل/ الرضاعة الطبيعية |  |  |  |  |  |

1. If you already took the vaccine, how did you decide to take it? / إذا كنت قد أخذت اللقاح بالفعل، فكيف قررت أخذه؟

My doctor/ gynecologist recommended it/ أوصى به طبيبي/ طبيب الأمراض النسائية

My family recommended it/ عائلتي أوصت به

I believe it is essential for me and my baby’s health/ أعتقد أنه ضروري لي ولصحة طفلي

I took it without being convinced/ أخذته دون أن أقتنع به

1. What are the reasons why you would take/took the COVID-19 vaccine? / ما هي الأسباب التي تجعلك/جعلتك تأخذين لقاح الكورونا؟
2. To protect myself from getting sick with COVID-19/ لحماية نفسي من الإصابة بفيروس الكورونا
3. To protect my baby/لحماية طفلي
4. To protect others in my family/لحماية الآخرين في عائلتي

To protect the community from getting sick with COVID-19/لحماية المجتمع من الإصابة بفيروس الكورونا

1. What are the reasons why you wouldn’t take the COVID-19 vaccine? / ما هي أسباب عدم أخذ لقاح كورونا؟
2. I have concerns about vaccine safety for myself/لدي مخاوف بشأن سلامة اللقاح لنفسي
3. I have concerns about vaccine safety for my pregnancy/ my baby/ لدي مخاوف بشأن سلامة اللقاح لحملي/ لطفلي
4. I have concerns about whether the vaccine would work to protect me from COVID-19/ لدي مخاوف ما إذا كان اللقاح سيعمل على حمايتي من كوفيد-19
5. I don’t think I need the vaccine/لا أعتقد أنني بحاجة للّقاح
